# Purinergic signaling as a basis of acupuncture-induced analgesia

**DOI:** 10.1007/s11302-020-09708-z

**Published:** 2020-06-23

**Authors:** Jin-Rong He, Shu-Guang Yu, Yong Tang, Peter Illes

**Affiliations:** 1 International Collaborative Centre on Big Science Plan for Purine Signalling, Chengdu University of Traditional Chinese Medicine, Chengdu, 610075 China; 2 Acupuncture & Chronobiology Key Laboratory of Sichuan Province, Chengdu, 610075 China; 3 Rudolf Boehm Institute for Pharmacology and Toxicology, University of Leipzig, 04107 Leipzig, Germany

**Keywords:** Acupuncture, Electroacupuncture, ATP, Adenosine, P2X receptors, P2Y receptors, P1/A1 receptors

## Abstract

This review summarizes experimental evidence indicating that purinergic mechanisms are causally involved in acupuncture (AP)-induced analgesia. Electroacupuncture (EAP) and manual AP release at pain-relevant acupoints ATP which may activate purinergic P2X receptors (Rs) especially of the P2X3 type situated at local sensory nerve endings (peripheral terminals of dorsal root ganglion [DRG] neurons); the central processes of these neurons are thought to inhibit via collaterals of ascending dorsal horn spinal cord neurons, pain-relevant pathways projecting to higher centers of the brain. In addition, during AP/EAP non-neuronal P2X4 and/or P2X7Rs localized at microglial cells of the CNS become activated at the spinal or supraspinal levels. In consequence, these microglia secrete bioactive compounds such as growth factors, cytokines, chemokines, reactive oxygen, and nitrogen species, which modulate the ascending neuronal pathways conducting painful stimuli. Alternatively, ATP released at acupoints by AP/EAP may be enzymatically degraded to adenosine, stimulating in loco presynaptic A1Rs exerting an inhibitory influence on the primary afferent fibers (the above mentioned pain-sensing peripheral terminals of DRG neurons) which thereby fail to conduct action potentials to the spinal cord dorsal horn. The net effect of the stimulation of P2X3, P2X4, P2X7, and A1Rs by the AP/EAP-induced release of ATP/adenosine at certain acupoints will be analgesia.

## Introduction

Acupuncture (AP) is a therapeutic manipulation of traditional Chinese medicine, known for the past 3000 to 4000 years [1, 2]. Nowadays, acupuncture refers to a family of procedures involving physical or chemical stimulation at acupoints using a variety of techniques. In this overview, we will deal with manual acupuncture (insertion of needles into the cutis/subcutis and moving/twisting them in regular intervals), electroacupuncture (EAP; stimulation of these needles by different frequencies of electrical current), and moxibustion (burning of cone-shaped preparations of moxa, made of dried mugworth above the acupoints) [3].

AP has been used for the treatment of a whole range of illnesses, with particularly good efficiency to relieve chronic painful conditions [4, 5]. Because the clinical effectiveness of AP in pain conditions is in spite of a lot of positive evidence still a much debated issue, it is particularly important to relay on experiments carried out on laboratory animals [2]. The data have to be evaluated with stringent statistical methods including comparison with a sufficient number of controls [6]. Animal experimentation is also able to clarify the analgesic mode of action of AP on the cellular and systemic levels with high reliability.

In order to approach AP from a natural scientific standpoint, it was important to find out that AP by stimulating sensory nerve terminals in cutaneous/subcutaneous and muscle tissue causes the release of a plethora of neurotransmitters (noradrenaline, serotonin, acetylcholine, glutamate, GABA) and neuropeptides (opioids, cholecystokinin, substance P, somatostatin) in the brain and spinal cord [7–11]. It was reported that especially the noradrenergic and serotonergic descending inhibitory fiber tracts originating in the locus coeruleus and raphe magnus nuclei, respectively, and terminating at enkephalinergic interneurons of the spinal cord dorsal horn are executors of AP-induced analgesia. However, the endogenous opioid systems of the brain utilizing β-endorphin and dynorphins are also intimately involved in pain relief by AP. Experiments in rodents and human volunteers demonstrated that the administration of the opioid antagonist naloxone blocked the effect of AP [2, 12], and in consequence, opioid peptides were released in the CNS [13, 14], probably in a frequency-dependent manner [7]. Analgesia caused by low frequency EAP stimulation was mediated by μ and/or δ opioid receptors (β-endorphin, enkephalins), whereas that caused by high frequency EAP stimulation was mediated by κ opioid receptors (dynorphins) [15]. Opioids were also liberated in inflamed tissue from lymphocytes, monocytes/macrophages, and granulocytes by EAP, activating specific receptors (Rs) at sensory nerve terminals to suppress nociception [4].

ATP within cells is well known as a universal energy currency. However, after being escaped/released into the extracellular space, ATP may function as a (co) transmitter in the nervous system and a general signaling molecule in the rest of the organism [16]. Enzymatic systems dephosphorylate ATP to ADP and eventually to adenosine, all of those compounds functioning as additional signaling molecules. Membrane receptors for ATP have been classified into two types, the ligand-gated cationic channel P2X (seven mammalian subtypes: P2X1, 2, 3, 4, 5, 6, 7) and the G protein-coupled P2Y receptors (eight mammalian subtypes: P2Y1, 2, 4, 6, 11, 12, 13, 14) [17–19]. Adenosine activates four types of P1 receptors termed A1, A2A, A2B, and A3.

More recently Geoffrey Burnstock advanced the hypothesis that ATP released by acupuncture needling may stimulate sensory nerve terminals and thereby complement the analgesic effect of opioid peptides [20]. P2XRs (P2X3, P2X4, P2X7), P2YRs (P2Y1), and P1/A1Rs have been reported to participate in this effect.

## Local release of ATP by AP needling

Cutaneous/subcutaneous mast cells are immune cells which play a role in anti-inflammatory responses, wound healing, angiogenesis, immune tolerance, and defense against pathogens. Due to their location, they are sensitive to mechanical stimulation from the external environment. Mast cells contain ATP which may be released as a result of AP needling [21, 22]. Four pieces of evidence support this notion: Firstly, mast cell-deficient rats (with *c-kit* gene mutant) exhibit less mechanical analgesia than their wild-type counterparts [22]. Secondly, mechanical stimuli lead to a rise in intracellular Ca^2+^ of mast cells and release ATP in a Ca^2+^-dependent manner [23]. Thirdly, non-specific P2R antagonists or specific P2X7 or P2Y13R antagonists attenuate the release of ATP from mast cells suggesting the occurrence of an ATP-induced ATP release [21]. Fourthly, the outflow of ATP and a whole range of ATP metabolites (ADP/AMP/adenosine) have been measured in the neighboring interstitium by a microdialysis probe after AP of the Zusanli point (ST36) of mice [24, 25]. The concentration increase of ATP rapidly declined to baseline after AP, whereas AMP, ADP, and adenosine remained significantly elevated [24]. It is important to note that ATP is enzymatically degraded by ecto-ATPases to AMP through the intermediary step ADP; AMP in turn is further dephosphorylated to adenosine by 5′-nucleotidase [26, 27]. Finally, adenosine is decomposed by adenosine deaminase to inosine, which has only minor effects at adenosine receptors. It has been appreciated only recently that in addition to 5′-nucleotidase, also a membrane-bound splice variant of prostatic acid phosphatase is widely distributed in the organism and has to be taken into account as an enzyme participating in adenosine production from AMP [28].

Another open question relates to the molecular mechanism that senses mechanical forces at the mast cell membrane and transduces it to trigger ATP release. Whole-cell patch-clamp recordings from the human leukemia mast cell line HMC-1 showed that mechanical stress caused a current which could be blocked by ruthenium red and SKF96365, two blockers of transient receptor potential vanilloid 2 (TRPV2) channels [29]. TRPV2 channels respond to mechanosensory stimulation and to noxious heat (> 52 °C) with the initiation of a nonselective cationic current. Apparently, activation of TRPV2 allowed extracellular Ca^2+^ to pass the mast cell membrane and to induce degranulation of stored constituents such as histamine, and ATP, degraded to adenosine. Then, these mast cell products caused analgesia, the first one probably via releasing β-endorphin into the cerebrospinal fluid [30] and the second one on its own right, by activating inhibitory A1Rs located at the peripheral terminals of DRG neurons (see below). Alternatively, it was suggested that the recently discovered mechanosensitive ion channel called “Piezo1” could be the immediate sensor of AP stimulation of the mast cell membrane [31].

## AP-induced analgesia by activation of adenosine A1Rs at sensory nerve terminals

A most interesting article reported that gentle manual rotation of an AP needle inserted into the Zusanli acupoint released adenosine into the tibialis anterior muscle/subcutis as measured by microdialysis and HPLC (see above; Ref. [24]). The concentration of adenosine increased approximately 24-fold and only slowly returned to the pre-AP value. Interestingly the local application of an A1R antagonist (2-chloro-N^6^-cyclopentyl-adenosine; CCPA) into the Zusanli acupoint inhibited mechanical and thermal allodynia, induced by the injection of complete Freund’s adjuvant (CFA) into the right paw [24, 32]. CFA is known to cause inflammatory pain. Experiments with CCPA in mice lacking A1Rs confirmed that A1R expression was necessary to record pain suppression. Spared injury to the sciatic nerve generated neuropathic pain which could also be relieved by the injection of CCPA into the ipsi- but not the contralateral Zusanli acupoint.

It was concluded that the relatively high extracellular concentrations of ATP metabolites compared with those of their mother compound likely represent the rapid enzymatic degradation of ATP by ecto-nucleotidases. Blockade of adenosine deaminase by deoxycoformycin increased the concentration of adenosine after AP and, as expected, elevated the analgesic potency of AP both in the inflammatory and neuropathic pain models.

Prostatic acid phosphatase (PAP) dephosphorylates AMP to adenosine. The injection of PAP into the Weizhong acupoint (BL40) in the poplitea fossa, located near to the Zusanli point, caused long-lasting analgesia towards mechanical and thermal painful stimulation in the CFA-treated hind paw of mice [33]. This was suggested to be due to an increase in the local concentration of adenosine and the subsequent stimulation of neuronal A1Rs within the acupoint. Antinociception by PAP could be transiently boosted with additional substrate (AMP) or transiently blocked with the A1R antagonist 8-cyclopentyl-1,3-dipropylxanthine (CPX). The strong analgesic effect of PAP was already previously documented in inflammatory and neuropathic pain models of mice after its intra-spinal injection [33].

## AP-induced analgesia by activation of P2XRs at sensory nerve terminals or at higher CNS centers

The results discussed above indicate that the effect of adenosine during AP is probably due to the local stimulation of A1Rs situated at the peripheral terminals of small and medium-sized dorsal root ganglion (DRG) neurons. By contrast, the mother compound of adenosine, ATP itself, is expected to cause pain by occupying P2XR types at the same sensory neurons (P2X3R, Refs. [34, 35]) or at neighboring macrophages (P2X4Rs, Ref. [36]; P2X7Rs, Refs. [37, 38]). In case of various cell-damaging, noxious stimuli, ATP is released/outpoured from the intracellular space either through the leaky cell membrane or by means of transporters pumping out ATP into the cellular interstitium, and then this ATP activates at lower concentrations P2X3 and/or P2X4 and at higher concentrations P2X7Rs [35].

However, during AP, nociception may turn into anti-nociception, when, for example, P2X3Rs desensitize on their long-lasting activation or P2X4/P2X7Rs release various bioactive molecules from macrophages which themselves block action potential generation at the terminals of DRG neurons. A still simpler explanation for the inverse analgesic effect of P2XRs would be that AP-induced impulses evoked in sensory fibers of the skin connect with interneurons to inhibit the neuronal pathways to the higher pain centers of the brain [20]. We will conclude from the available data sets that this latter mode of action is highly possible.

### P2X3 receptors

Neuropathic pain in rats was induced by constricting one sciatic nerve with a ligature (chronic constriction injury; CCI) [39–41]. In these rats, the paw withdrawal threshold (PWT) to pressure and the paw withdrawal latency (PWL) to radiant heat have been measured as two parameters of hyperalgesia and allodynia, respectively. During the 14 days of observation time, both parameters gradually declined and then leveled off at a minimum value starting with the 7th day. At this time point, EAP was applied to ipsi- or contralateral acupoints (Zusanli or Yanglinquan [GB34]) causing a gradual and moderate reversal of the neuropathy symptoms. The equal sensitivity to ipsi- and contralateral EAP suggested that the treatment acted at the spinal or supraspinal levels rather than at the peripheral terminals of DRG neurons.

Patch-clamp measurements from acutely dissociated DRG neurons showed that the P2X1-3R selective ATP analogue α,β-methylene ATP (α,β-meATP) initiated larger current amplitudes in preparations taken from rats which underwent CCI, than in their non-operated counterparts [39–41]. The potentiation of the α,β-meATP currents by CCI was partially counteracted by EAP treatment. Quantitative reverse transcription polymerase chain reaction (RT-PCR), in situ hybridization, quantitative immunohistochemistry, and western blotting all showed an increased appearance of the P2X3R mRNA/protein in DRG neurons and the appropriate segments of the spinal cord dorsal horn after CCI to the unilateral sciatic nerve [40, 42]. EAP moderately counteracted this increase, without any side preference.

Diabetic neuropathy was modeled by feeding rats with a high-fat and high-sugar diet for 5 weeks plus injecting once streptozocin [43]. Streptozocin injection alone would primarily damage the pancreas only (diabetes type 1), while in combination with the diet used, it caused long-lasting hyperglycemia and histological injury to the sciatic nerve as well (diabetes type 2). EAP with a frequency of 2 Hz normalized the previously increased PWT and plasma membrane P2X3 protein level measured by western blotting but failed to improve the destructive ultrastructural changes of the sciatic nerve. Hence, anti-nociception was achieved without improving the underlying condition of neuropathic damage.

When the PWT and PWL were measured with mechanical pressure and heat, respectively, on the CFA-treated hind paw of rats, EAP applied to Zusanli and BL60 (Kunlun acupoint) exerted analgesia in this inflammatory pain model [44, 45]. It was noted that 100 Hz EAP stimulation had a stronger effect than stimulation with 2 Hz. In addition, EAP reversed the increased P2X3R-immunoreactivity and protein expression in DRGs, as measured by quantitative immunohistochemistry and western blotting, respectively [44, 45].

Visceral pain was also found to react to EAP. A model of the painful irritable bowel syndrome was generated in few day old rats by inflating a balloon in their terminal colon/rectum twice daily for 2 weeks in total [2, 46, 47]. These rats developed hypersensitivity to bowel distension within a further period of 6 weeks elapsing without any pressure stimulation. An arbitrary withdrawal reflex score was used to determine the intensity of pain reaction caused by subsequent colorectal distension. In pressure-treated rats, the pain withdrawal score increased in an EAP reversible manner. The expression levels of P2X3R mRNA and protein in DRGs also responded with increase and decrease to balloon distension and EAP, respectively.

### P2X4 receptors

Relatively few data are available about P2X4Rs. It was reported that EAP to Huantiao acupoint (GB30) for 14 days increased the PWT in rats with CCI [48]. The elevated expression of P2X4R and interferon-γ (IFN-γ) mRNA and protein in the spinal cord of CCI rats was suppressed by EAP. It was concluded that EAP ameliorated tactile allodynia after peripheral nerve injury by downregulating excessive production of IFN-γ which is a stimulus for the expression of P2X4Rs in spinal cord microglia. Otherwise, after CCI, microglia (the resident macrophages of the CNS) would release on ATP-induced activation brain-derived neurotrophic factor (BDNF), causing an altered transmembrane anion gradient in a subpopulation of dorsal horn lamina 1 neurons. This results in the downregulation of the neuronal chloride transporter KCC2 and the subsequent conversion of GABA_A_ and glycin-R-mediated inhibition to excitation [49]. The enhanced excitability of lamina 1 neurons would lead to the clinical symptoms of neuropathic pain.

Another report focused on visceral hypersensitivity after colorectal distension in rats indicating that EAP at the Shangjuxu (ST37) and Tianshu (ST25) acupoints not only markedly decreased abdominal withdrawal reflex scores in rats with visceral hypersensitivity, but in addition P2X4R immunoreactivity in colon and spinal cord was decreased after EAP [50]. Both data sets suggested that CCI-induced stimulation of IFN-γ production acts via microglial P2X4R upregulation to increase the neuronal activity in the ascending pain pathways projecting to higher brain centers. EAP counteracted this effect.

### P2X7 receptors

*P2X4* and *P2X7* genes in humans are located on chromosome 12 in close proximity, indicating tight relationship in origin and function [51]. The overlapping expression of the receptor proteins has been documented especially in peripheral macrophages and microglia [51, 52]. The reason for the co-expression may be the involvement of both receptors in inflammatory processes. Whereas P2X4Rs stimulate the release of BDNF from microglia, P2X7Rs participate in the secretion of inflammatory cytokines, chemokines, proteases, reactive oxygen, and nitrogen species from activated microglia/macrophages. No wonder that this receptor is intimately involved in innate immunology and all types of pain reactions with an inflammatory component.

CCI or the intrathecal injection of the prototypic P2X7R agonist dibenzoyl-ATP (Bz-ATP) caused pain which was sensitive to EAP applied to the Huantiao acupoint [53]. This manipulation at the same time suppressed the overexpression of the two inflammatory cytokines, interleukin-1β (IL-1β) and IL-18. Further, in the neck incision pain model, both the levels of ATP and the expression of P2X7R mRNA and protein were upregulated in the spinal cord [54]. Both effects could be antagonized by EAP applied to the acupoints Zusanli and Yanglinquan. Eventually, moxibustion treatment at Dachangsu acupoint (BL25) decreased the abdominal reflex scores and the upregulation of P2X7Rs in DRGs after repeated balloon distension [55]. All these results confirm that microglial P2X7Rs in the spinal cord are involved in different pain modalities and that EAP is able to alleviate neuropathic, traumatic, and visceral pain probably via decreasing the secretion of pro-inflammatory molecules from spinal microglia.

### P2X3/ASIC3 cognate receptors

Protons activate pain-sensing nociceptors located at primary afferent fibers (the peripheral terminals of DRG neurons) which transmit information to the spinal cord and eventually to higher brain centers [38]. Acid sensing ion channel 3 (ASIC3) responds to moderate local tissue acidification with an inward Na^+^ current causing depolarization of sensory nerve terminals [56]. Although the amino acid composition of ASIC3 and P2X3R-channels is different, their overall structure (composition of three protein subunits) and ion conductive pathways are similar. Recently it has been suggested that ASIC3 and P2X3R subunits do not form a heteromeric channel but tightly associate with each other to constitute a protein complex, mediating unidirectional inhibition [57]. In accordance with this idea, the selective ASIC3 antagonists APETx2 and Ugr9-1 both prevented the painful reaction caused by the injection of slightly acidic medium (pH 6.0) into the hind paw [58]. By contrast, this nociceptive reaction was not altered by the highly selective P2X3R antagonist A-317491. According to expectations, both APETx2 and A-317491 interfered with the effects of protons and α,β-meATP, respectively.

After having confirmed the existence of an ASIC3/P2X3 “cognate” receptor by measuring the thermal PWL, when a laser beam was directed to the hind limb of rats, the effects of EAP and moxibustion were investigated on acidic and purinergically initiated nociceptive hypersensitivity [58]. During acute thermal pain and CFA-induced inflammatory pain, ASIC3-TRPV1 channels and P2X3-P2X7 receptors were activated by protons and exogenous α,β-meATP or endogenously released ATP, respectively. TRPV1 channels respond to pungent vanilloids, protons, and heat (> 42 °C) with conduction of a nonselective cationic current. It was found that low-threshold acidic pain (injection of pH 6.0 medium) mediated by the activation of ASIC3-TRPV1 channels was prevented by EAP/moxibustion, while high-threshold acidic pain (injection of pH 4.0 medium) mediated exclusively by the activation of TRPV1 channels was not. Further, EAP and moxibustion prevented nociception mediated by both types of purinergic receptors.

### P2Y1 receptors

A definite weakness of the available scientific literature is that there exist only few investigations on the involvement of the ATP/ADP-sensitive P2YRs in AP-induced analgesia. At least for P2Y1Rs, some data support an analgesic effect by the blockade of the N-type voltage-sensitive Ca^2+^ current in DRG neurons [59]. This may lead to a decreased release of the nociceptive transmitter glutamate from layer 1 sensory neurons in the spinal cord dorsal horn and thereby interrupt the synaptic contact to higher areas of the brain. Alternatively, a negative interaction between P2Y1Rs and the pain-causing TRPV1 channels may also result in analgesia [60].

However, still more findings support the notion that a number of P2Y receptor-types (P2Y1, P2Y6, P2Y11, P2Y12, P2Y13) boost painful sensation and their blockade by selective antagonists has an analgesic effect [2, 6]. While the reason for this apparent controversy is unknown, consonant with the existence of pain-mediating P2YRs , EAP was reported to inhibit visceral hypersensitivity caused by the intracolonic injection of acetic acid as a model of irritable bowel syndrome [61]. In these experiments, both a selective antagonist of P2Y1Rs and an antagonist of the mitogen-activated protein kinase/extracellular-regulated kinase 1 (MAPK/ERK) decreased the intensity of pain. The analgesia induced by EAP simultaneously with the downregulation of the astrocytic marker glial fibrillary acidic protein (GFAP) and P2Y1R immunoreactivities led to the conclusion that EAP depresses visceral hypersensitivity by inhibiting P2Y1Rs and their downstream signaling via the MAPK/ERK pathway in astrocytes. A strong argument for the participation of astrocytes and their P2Y1Rs in EAP-induced analgesia was supplied by experiments which documented the blockade of this analgesic reaction by the intrathecal infusion of the selective astrocytic neurotoxin, fluorocitrate.

In this case, not the EAP-induced local release of ATP/ADP was supposed to be involved but that of a bioactive molecule downregulating P2Y1R activity. Astrocytes on their behalf may cause neuronal effects indirectly by modifications in K^+^ uptake and redistribution, Cl^−^ and water fluxes, Na^+^/Ca^2+^ or Na^+^/HCO_3_^−^ exchange, neurotransmitter uptake, etc. [62]. Furthermore, astrocytes may release gliotransmitters by vesicular Ca^2+^-dependent mechanisms and by connexin/pannexin (hemi)channels and other non-exocytotic pathways, modifying neuronal functions.

### P2Y13 receptors

P2Y13Rs via the stimulation of pro-inflammatory cytokine secretion (IL-1β, IL-6) and the activation of the p38 MAPK pathway have been repeatedly reported to be involved in the development/manifestation of neuropathic pain [63, 64]. In this respect, it is important to refer to findings already presented previously (see Section “Local release of ATP by AP needling”). Paracrine P2Y13Rs have been shown to facilitate the mechanosensitive release of ATP from the mast cell-like rat basophilic leukemia cell line RBL-2HR, in a manner inhibited by the selective P2Y13R antagonist MRS 2211 [21]. Unfortunately, the interaction between MRS2211 and AP-induced analgesia was not investigated in this otherwise fascinating study, and therefore, no conclusion can be drawn on the involvement of P2Y13Rs in the mode of action of AP.

## Conclusion

**Fig. 1:**
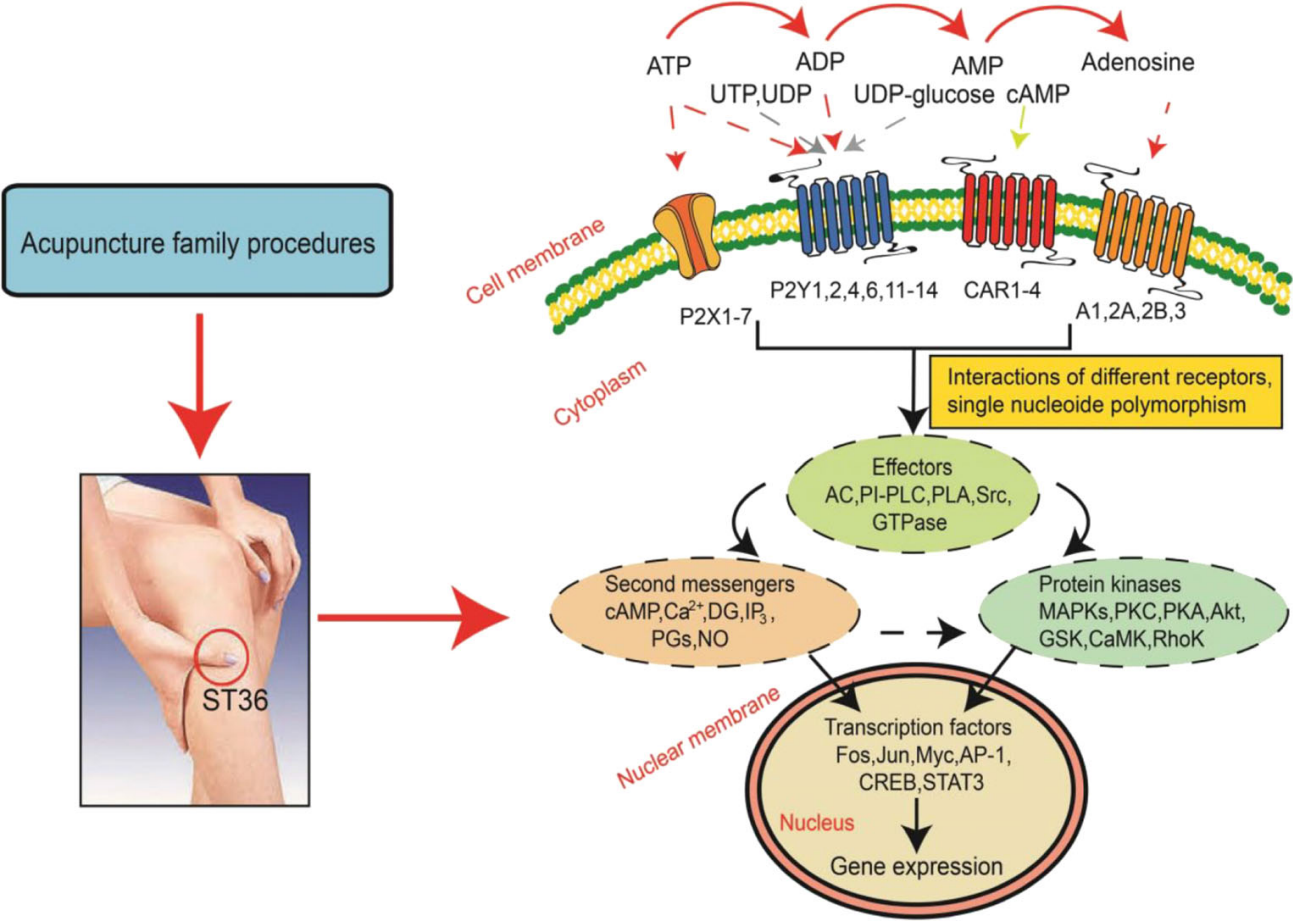
Acupuncture family procedures applied to certain acupoints alleviate pain. These procedures release ATP locally which is sequentially degraded by ecto-ATPases to ADP/AMP and then by 5′-nucleotidase to adenosine. ATP stimulates P2XRs of the P2X3, P2X4, and P2X7 subtypes, while adenosine stimulates P1Rs of the A1 subtype. Further complexity may be added to AP-induced analgesia via interactions between these receptors in the plasma membrane or interactions between their downstream signaling mechanisms. CAR1–4, four subtypes of G protein-linked cAMP receptors; AC, adenylcyclase; PI-PLC, phosphatidylinositol-bisphosphate phosphodiesterase; PLA, phospholipase A; Src, Src tyrosin kinase; cAMP, cyclic adenosine monophosphate; DG, diacylglycerol; IP3, inositoltrisphosphate; PGs, prostaglandins; MAPKs, mitogen-activated protein kinases; PKC, protein kinase C; PKA, protein kinase A; Akt, protein kinase B; GSK, GS kinase; CaMK, Ca^2+^/calmodulin-dependent protein kinase; RhoK, Rho kinase; CREB, cAMP response element-binding protein; STAT3, signal transducer and activator of transcription 3. Reproduced by permission from Ref. [2]

Because of the controversial discussion of the efficiency of AP in painful conditions, it is most important to rely on experiments carried out on laboratory animals subsequently evaluated by stringent statistical methods. The inspection of such experimental data supplies strong evidence for the involvement of purinergic mechanisms in AP-induced analgesia (Fig. 1). ATP and its enzymatic degradation products ADP/AMP/adenosine and UTP/UDP, UDP-glucose, and cAMP may stimulate a range of purine and pyrimidine receptors. As discussed in this overview, hitherto only P2X3, P2X4, P2X7, and A1Rs have been shown to verifiably participate in AP-induced analgesia. However, interactions between the downstream signaling mechanisms of these receptors may also occur, adding further complexity to the present, possibly oversimplified picture. Eventually, purinergic mechanisms may complement effects due to the release of opioid peptides by AP in the CNS and peripheral inflamed tissues.

## Abbreviations

AP: Acupuncture
ASIC3: Acid-sensing ion channel 3
Bz-ATP: Dibenzoyl-ATP
CCI: Chronic constriction injury
CCPA: 2-Chloro-N^6^-cyclopentyl-adenosine
CFA: Complete Freund’s adjuvant
CPX: 8-Cyclopentyl-1,3-dipropylxanthine
DRG: Dorsal root ganglion
EAP: Electroacupuncture
fEPSP: Field excitatory postsynaptic potential
GABA_A_-R: γ-Aminobutyric acid receptor A
IFN-γ: Interferon-γ
IL-1β: Interleukin-1β
PAP: Prostatic acid phosphatase
P2XR: Purinergic ligand-gated cationic channel
P2YR: Purinergic G protein-coupled receptor
R: Receptor
TRPV1: Transient receptor potential vanilloid 1
α,β-meATP: α,β-methylene ATP

## References

[1] White A, Ernst E (2004). A brief history of acupuncture. Rheumatology (Oxford).

[2] Tang Y, Yin HY, Rubini P, Illes P (2016). Acupuncture-induced analgesia: a neurobiological basis in purinergic signaling. Neuroscientist.

[3] Fu SF, KunW, Zeng XX, Zhang L, Cheng CW, Song L, Zhong LLD, Lin J, Wang YY, Shang HC, Bian ZX, CARC Group (2016) Urgent need to improve the quality of case report in traditional Chinese medicine: assessment on reporting quality of 3,417 cases. Chin J Integr Med 22:473–480 10.1007/s11655-015-2250-y 26801486

[4] Zhang R, Lao L, Ren K, Berman BM (2014). Mechanisms of acupuncture-electroacupuncture on persistent pain. Anesthesiology.

[5] Vickers AJ, Linde K (2014). Acupuncture for chronic pain. JAMA.

[6] Zhang X, Li G (2019). P2Y receptors in neuropathic pain. Pharmacol Biochem Behav.

[7] Han JS (2003). Acupuncture: neuropeptide release produced by electrical stimulation of different frequencies. Trends Neurosci.

[8] Li P, Longhurst JC (2010). Neural mechanism of electroacupuncture's hypotensive effects. Auton Neurosci.

[9] Yang ES, Li PW, Nilius B, Li G (2011). Ancient Chinese medicine and mechanistic evidence of acupuncture physiology. Pflugers Arch.

[10] Chen S, Wang S, Rong P, Wang J, Qiao L, Feng X, Liu J, Zhang J (2014) Acupuncture for visceral pain: neural substrates and potential mechanisms. Evid Based Complement Alternat Med 2014:609594 10.1155/2014/609594 PMC4295157 25614752

[11] Kim W, Kim SK, Min BI (2013). Mechanisms of electroacupuncture-induced analgesia on neuropathic pain in animal model. Evid Based Complement Alternat Med.

[12] Pomeranz B, Chiu D (1976). Naloxone blockade of acupuncture analgesia: endorphin implicated. Life Sci.

[13] Mayer DJ, Price DD, Rafii A (1977). Antagonism of acupuncture analgesia in man by the narcotic antagonist naloxone. Brain Res.

[14] Cheng RS, Pomeranz B (1979). Electroacupuncture analgesia could be mediated by at least two pain-relieving mechanisms; endorphin and non-endorphin systems. Life Sci.

[15] Chen XH, Han JS (1992). All three types of opioid receptors in the spinal cord are important for 2/15 Hz electroacupuncture analgesia. Eur J Pharmacol.

[16] Burnstock G (1972). Purinergic nerves. Pharmacol Rev.

[17] Abbracchio MP, Burnstock G (1994). Purinoceptors: are there families of P2X and P2Y purinoceptors?. Pharmacol Ther.

[18] von Kugelgen I, Hoffmann K (2016). Pharmacology and structure of P2Y receptors. Neuropharmacology.

[19] Jarvis MF, Khakh BS (2009). ATP-gated P2X cation-channels. Neuropharmacology.

[20] Burnstock G (2009). Acupuncture: a novel hypothesis for the involvement of purinergic signalling. Med Hypotheses.

[21] Shen D, Shen X, Schwarz W, Grygorczyk R, Wang L (2020) P2Y13 and P2X7 receptors modulate mechanically induced adenosine triphosphate release from mast cells. Exp Dermatol 29:499–508 10.1111/exd.14093 32155290

[22] Cui X, Liu K, Xu D, Zhang Y, He X, Liu H, Gao X, Zhu B (2018). Mast cell deficiency attenuates acupuncture analgesia for mechanical pain using c-kit gene mutant rats. J Pain Res.

[23] Yao W, Yang H, Yin N, Ding G (2014). Mast cell-nerve cell interaction at acupoint: modeling mechanotransduction pathway induced by acupuncture. Int J Biol Sci.

[24] Goldman N, Chen M, Fujita T, Xu Q, Peng W, Liu W, Jensen TK, Pei Y, Wang F, Han X, Chen JF, Schnermann J, Takano T, Bekar L, Tieu K, Nedergaard M (2010). Adenosine A1 receptors mediate local anti-nociceptive effects of acupuncture. Nat Neurosci.

[25] Takano T, Chen X, Luo F, Fujita T, Ren Z, Goldman N, Zhao Y, Markman JD, Nedergaard M (2012). Traditional acupuncture triggers a local increase in adenosine in human subjects. J Pain.

[26] Yegutkin GG (2008). Nucleotide- and nucleoside-converting ectoenzymes: important modulators of purinergic signalling cascade. Biochim Biophys Acta.

[27] Zimmermann H, Zebisch M, Strater N (2012). Cellular function and molecular structure of ecto-nucleotidases. Purinergic Signal.

[28] Zimmermann H (2009). Prostatic acid phosphatase, a neglected ectonucleotidase. Purinergic Signal.

[29] Zhang D, Spielmann A, Wang L, Ding G, Huang F, Gu Q, Schwarz W (2012) Mast-cell degranulation induced by physical stimuli involves the activation of transient-receptor-potential channel TRPV2. Physiol Res 61:113–124 10.33549/physiolres.932053 21574765

[30] Huang M, Wang X, Xing B, Yang H, Sa Z, Zhang D, Yao W, Yin N, Xia Y, Ding G (2018). Critical roles of TRPV2 channels, histamine H1 and adenosine A1 receptors in the initiation of acupoint signals for acupuncture analgesia. Sci Rep.

[31] Wei L, Mousawi F, Li D, Roger S, Li J, Yang X, Jiang LH (2019). Adenosine triphosphate release and P2 receptor signaling in Piezo1 channel-dependent mechanoregulation. Front Pharmacol.

[32] Zylka MJ (2011). Pain-relieving prospects for adenosine receptors and ectonucleotidases. Trends Mol Med.

[33] Hurt JK, Zylka MJ (2012). PAPupuncture has localized and long-lasting antinociceptive effects in mouse models of acute and chronic pain. Mol Pain.

[34] Chizh BA, Illes P (2001). P2X receptors and nociception. Pharmacol Rev.

[35] Wirkner K, Sperlagh B, Illes P (2007). P2X3 receptor involvement in pain states. Mol Neurobiol.

[36] Tsuda M, Inoue K, Salter MW (2005). Neuropathic pain and spinal microglia: a big problem from molecules in "small" glia. Trends Neurosci.

[37] Sperlagh B, Illes P (2014). P2X7 receptor: an emerging target in central nervous system diseases. Trends Pharmacol Sci.

[38] Burnstock G (2016). Purinergic mechanisms and pain. Adv Pharmacol.

[39] Cheng RD, Tu WZ, Wang WS, Zou EM, Cao F, Cheng B, Wang JZ, Jiang YX, Jiang SH (2013). Effect of electroacupuncture on the pathomorphology of the sciatic nerve and the sensitization of P2X3 receptors in the dorsal root ganglion in rats with chronic constrictive injury. Chin J Integr Med.

[40] Tu WZ, Cheng RD, Cheng B, Lu J, Cao F, Lin HY, Jiang YX, Wang JZ, Chen H, Jiang SH (2012). Analgesic effect of electroacupuncture on chronic neuropathic pain mediated by P2X3 receptors in rat dorsal root ganglion neurons. Neurochem Int.

[41] Wang WS, Tu WZ, Cheng RD, He R, Ruan LH, Zhang L, Gong YS, Fan XF, Hu J, Cheng B, Lai YP, Zou EM, Jiang SH (2014). Electroacupuncture and A-317491 depress the transmission of pain on primary afferent mediated by the P2X3 receptor in rats with chronic neuropathic pain states. J Neurosci Res.

[42] Liang Y, Gu Y, Shi R, Li G, Chen Y, Huang LM (2019). Electroacupuncture downregulates P2X3 receptor expression in dorsal root ganglia of the spinal nerve-ligated rat. Mol Pain.

[43] Zhou YF, Ying XM, He XF, Shou SY, Wei JJ, Tai ZX, Shao XM, Liang Y, Fang F, Fang JQ, Jiang YL (2018). Suppressing PKC-dependent membrane P2X3 receptor upregulation in dorsal root ganglia mediated electroacupuncture analgesia in rat painful diabetic neuropathy. Purinergic Signal.

[44] Fang JQ, Du JY, Fang JF, Xiao T, Le XQ, Pan NF, Yu J, Liu BY (2018) Parameter-specific analgesic effects of electroacupuncture mediated by degree of regulation TRPV1 and P2X3 in inflammatory pain in rats. Life Sci 200:69–80 10.1016/j.lfs.2018.03.028 29550358

[45] Xiang X, Wang S, Shao F, Fang J, Xu Y, Wang W, Sun H, Liu X, du J, Fang J (2019) Electroacupuncture stimulation alleviates CFA-induced inflammatory pain via suppressing P2X3 expression. Int J Mol Sci 20(13) 10.3390/ijms20133248 PMC6651287 31269659

[46] Weng ZJ, Wu LY, Zhou CL, Dou CZ, Shi Y, Liu HR, Wu HG (2015). Effect of electroacupuncture on P2X3 receptor regulation in the peripheral and central nervous systems of rats with visceral pain caused by irritable bowel syndrome. Purinergic Signal.

[47] Weng Z, Wu L, Lu Y, Wang L, Tan L, Dong M, Xin Y (2013). Electroacupuncture diminishes P2X2 and P2X3 purinergic receptor expression in dorsal root ganglia of rats with visceral hypersensitivity. Neural Regen Res.

[48] Chen XM, Xu J, Song JG, Zheng BJ, Wang XR (2015). Electroacupuncture inhibits excessive interferon-gamma evoked up-regulation of P2X4 receptor in spinal microglia in a CCI rat model for neuropathic pain. Br J Anaesth.

[49] Coull JA, Beggs S, Boudreau D, Boivin D, Tsuda M, Inoue K, Gravel C, Salter MW, Koninck YD (2005) BDNF from microglia causes the shift in neuronal anion gradient underlying neuropathic pain. Nature 438:1017–1021 10.1038/nature04223 16355225

[50] Guo X, Chen J, Lu Y, Wu L, Weng Z, Yang L, Xin Y, Lin X, Liang Y, Fang J (2013). Electroacupuncture at He-mu points reduces P2X4 receptor expression in visceral hypersensitivity. Neural Regen Res.

[51] Suurvali J, Boudinot P, Kanellopoulos J, Ruutel BS (2017). P2X4: a fast and sensitive purinergic receptor. Biom J.

[52] Dubyak GR (2007). Go it alone no more--P2X7 joins the society of heteromeric ATP-gated receptor channels. Mol Pharmacol.

[53] Xu J, Chen XM, Zheng BJ, Wang XR (2016). Electroacupuncture relieves nerve injury-induced pain hypersensitivity via the inhibition of spinal P2X7 receptor-positive microglia. Anesth Analg.

[54] Gao YH, Li CW, Wang JY, Tan LH, Duanmu CL, Jing XH, Chang XR, Liu JL (2017). Effect of electroacupuncture on the cervicospinal P2X7 receptor/fractalkine/CX3CR1 signaling pathway in a rat neck-incision pain model. Purinergic Signal.

[55] Liu S, Shi Q, Zhu Q, Zou T, Li G, Huang A, Wu B, Peng L, Song M, Wu Q, Xie Q, Lin W, Xie W, Wen S, Zhang Z, Lv Q, Zou L, Zhang X, Ying M, Li G, Liang S (2015). P2X7 receptor of rat dorsal root ganglia is involved in the effect of moxibustion on visceral hyperalgesia. Purinergic Signal.

[56] Wemmie JA, Taugher RJ, Kreple CJ (2013). Acid-sensing ion channels in pain and disease. Nat Rev Neurosci.

[57] Stephan G, Huang L, Tang Y, Vilotti S, Fabbretti E, Yu Y, Nörenberg W, Franke H, Gölöncsér F, Sperlágh B, Dopychai A, Hausmann R, Schmalzing G, Rubini P, Illes P (2018). The ASIC3/P2X3 cognate receptor is a pain-relevant and ligand-gated cationic channel. Nat Commun.

[58] Zhang Y, Huang L, Kozlov SA, Rubini P, Tang Y, Illes P (2020). Acupuncture alleviates acid- and purine-induced pain in rodents. Br J Pharmacol.

[59] Gerevich Z, Borvendeg SJ, Schroder W, Franke H, Wirkner K, Nörenberg W, Fürst S, Gillen C, Illes P (2004) Inhibition of N-type voltage-activated calcium channels in rat dorsal root ganglion neurons by P2Y receptors is a possible mechanism of ADP-induced analgesia. J Neurosci 24:797–807 10.1523/JNEUROSCI.4019-03.2004 PMC6729814 14749424

[60] Stanchev D, Blosa M, Milius D, Gerevich Z, Rubini P, Schmalzing G, Eschrich K, Schaefer M, Wirkner K, Illes P (2009). Cross-inhibition between native and recombinant TRPV1 and P2X3 receptors. Pain.

[61] Zhao J, Li H, Shi C, Yang T, Xu B (2020). Electroacupuncture inhibits the activity of astrocytes in spinal cord in rats with visceral hypersensitivity by inhibiting P2Y1 receptor-mediated MAPK/ERK signaling pathway. Evid Based Complement Alternat Med.

[62] Illes P, Burnstock G, Tang Y (2019). Astroglia-derived ATP modulates CNS neuronal circuits. Trends Neurosci.

[63] Kobayashi K, Yamanaka H, Yanamoto F, Okubo M, Noguchi K (2012). Multiple P2Y subtypes in spinal microglia are involved in neuropathic pain after peripheral nerve injury. Glia.

[64] Zhou R, Xu T, Liu X, Chen Y, Kong D, Tian H, Yue M, Huang D, Zeng J (2018) Activation of spinal dorsal horn P2Y13 receptors can promote the expression of IL-1β and IL-6 in rats with diabetic neuropathic pain. J Pain Res 11:615–628 10.2147/JPR.S154437 PMC5877493 29628771

